# Understanding disciplinary perspectives: a framework to develop skills for interdisciplinary research collaborations of medical experts and engineers

**DOI:** 10.1186/s12909-024-05913-1

**Published:** 2024-09-13

**Authors:** Sophie van Baalen, Mieke Boon

**Affiliations:** 1 Department of Philosophy, University of Twente, Enschede, The Netherlands; 2 Rathenau Instituut, Den Haag, The Netherlands

**Keywords:** Adaptive expertise, Interdisciplinary expertise, Metacognitive skills, Higher-order cognitive abilities, Epistemology, Problem-solving, Reflection, Disciplinary perspectives, Medical technology

## Abstract

**Background:**

Health professionals need to be prepared for interdisciplinary research collaborations aimed at the development and implementation of medical technology. Expertise is highly domain-specific, and learned by being immersed in professional practice. Therefore, the approaches and results from one domain are not easily understood by experts from another domain. Interdisciplinary collaboration in medical research faces not only institutional, but also cognitive and epistemological barriers. This is one of the reasons why interdisciplinary and interprofessional research collaborations are so difficult. To explain the cognitive and epistemological barriers, we introduce the concept of *disciplinary perspectives*. Making explicit the disciplinary perspectives of experts participating in interdisciplinary collaborations helps to clarify the specific approach of each expert, thereby improving mutual understanding.

**Method:**

We developed a framework for making disciplinary perspectives of experts participating in an interdisciplinary research collaboration explicit. The applicability of the framework has been tested in an interdisciplinary medical research project aimed at the development and implementation of diffusion MRI for the diagnosis of kidney cancer, where the framework was applied to analyse and articulate the disciplinary perspectives of the experts involved.

**Results:**

We propose a general framework, in the form of a series of questions, based on new insights from the philosophy of science into the epistemology of interdisciplinary research. We explain these philosophical underpinnings in order to clarify the cognitive and epistemological barriers of interdisciplinary research collaborations. In addition, we present a detailed example of the use of the framework in a concrete interdisciplinary research project aimed at developing a diagnostic technology. This case study demonstrates the applicability of the framework in interdisciplinary research projects.

**Conclusion:**

Interdisciplinary research collaborations can be facilitated by a better understanding of how an expert’s disciplinary perspectives enables and guides their specific approach to a problem. Implicit disciplinary perspectives can and should be made explicit in a systematic manner, for which we propose a framework that can be used by disciplinary experts participating in interdisciplinary research project. Furthermore, we suggest that educators can explore how the framework and philosophical underpinning can be implemented in HPE to support the development of students’ interdisciplinary expertise.

## Background

Expertise is highly domain-specific, and learned by being immersed in professional practice [1]. However, today’s rapidly evolving health care systems require clinicians who are capable of meeting complex challenges [2], which often requires interdisciplinary and interprofessional collaborations between experts from distinct disciplines.^1^ With the increasingly central role of innovative medical technologies in many medical specialties [3], health professionals will presumable participate in interdisciplinary and interprofessional research collaborations. But interprofessional and interdisciplinary research collaborations are notoriously difficult (e.g., [4–7]). Boon et al. (2019) argue that the complexity of current medical practices requires *interdisciplinary expertise*, which is an extension of *adaptive expertise* [8]. Interdisciplinary expertise involves the ability to understand the role of *disciplinary perspectives*.

In this paper, we combine insights from the *philosophy of science* on disciplinary perspectives and practice experience from an interdisciplinary medical research project aimed at the development and implementation of diffusion MRI for the diagnosis of kidney cancer. Based on these insights and practice experience, we propose a framework for mitigating cognitive and epistemological barriers caused by different disciplinary perspectives. In addition, we present a detailed example of the use of the framework to analyse and explain the experts’ disciplinary perspectives in the aforementioned interdisciplinary research project aimed at developing a diagnostic technology. This case study demonstrates the use of the framework in interdisciplinary research projects. The framework can be used by health professionals to facilitate their interdisciplinary research projects, by analysing and explaining their disciplinary perspectives.

### Interdisciplinary research

To address the barriers to interdisciplinary research, various authors have developed analytical frameworks to guide the research process and help disciplinary experts understand what it takes to execute projects together with experts from other disciplines [9–12]. Menken et al. (2016), for example, provide a method for interdisciplinary research that is much similar to the traditional empirical cycle, including steps such as “identify problem or topic,” “formulate preliminary research questions,” “data collection” and “draw conclusions” [11]. Other frameworks describe which steps need to be taken in the interdisciplinary research *process*. In the literature on *team science*, several authors also aim to provide a better understanding of the process of interdisciplinary research. For example, Hasan et al. (2023) focuses on the ‘micro’ layers of the team science ecosystem proposed by Stokols et al. (2019) – the layer of individual team members collaborating in interdisciplinary research projects [13, 14]. From their analysis of an online collaborations between early academics from different fields, they provide insights into common issues in interdisciplinary research and methods for dealing with them. By applying their framework from the start of the interdisciplinary research process, they argue, interdisciplinary capture [15] can be avoided.

Although the aforementioned frameworks provide valuable guidance on the *process* of interdisciplinary collaboration, they do not address the deeper cognitive and epistemological challenges of interdisciplinary research collaboration [5, 16], which is the objective of our contribution. A crucial assumption in current frameworks seems to be that interdisciplinary research collaboration is learned by doing, and that the *integration* of different disciplines will automatically follow.^2^ In our view, however, the *integration* of different disciplines is both crucial and one of the most challenging aspects of interdisciplinary research collaboration. In previous work we have argued that the inherent *cognitive* and *epistemological* (knowledge-theoretical) challenges of integration have been neglected by most authors providing models for interdisciplinary research [8]. In this paper, our focus is therefore on challenges of *using and producing knowledge* in interdisciplinary research collaborations that aim at solving complex real-world problems. Examples are collaborations between distinct medical specialists in the diagnosis and treatment of a specific patient (e.g., an oncologist and radiologist), but also collaborations between medical experts and biomedical engineers aimed at innovative medical technology for clinical uses. In this paper, we focus on *inter*disciplinary research projects, in which two or more academic fields are integrated to solve real-world problems, and not on *trans*disciplinary projects in which one or more academic fields are integrated with expertise from outside of academia such as policy-making or practice.^3^

The challenge of *interdisciplinary research collaborations* aimed at solving a shared problem is that each expert is guided by his/her own *disciplinary perspective.* However, the results produced by experts from different disciplines, although internally coherent, are not mutually coherent, so that they are not easily integrated. Furthermore, approaches and results understood within a contributing disciplinary perspective are not easily understood by experts specialised in other disciplinary perspectives, even though each expert aims to contribute to the same problem.

In short, the way in which experts use and produce knowledge is guided by the disciplinary perspective typical of their own practice. But experts are often unaware of having a disciplinary perspective. We argue that this is an obstacle to participating in *interdisciplinary research collaborations* focused on *using and producing knowledge* for *complex problem-solving*. Moreover, disciplinary perspectives are often considered impenetrable —as they are acquired *by doing*— which makes dealing with the disciplinary perspective of other experts a difficult learning objective. In this paper, we defend that disciplinary perspectives can be made explicit in a systematic manner, and that their role in ‘how experts in a specific discipline use and produce knowledge’ can thus be made understandable for experts and students in both their own and other disciplines.

To this end, we have developed a framework, based on new insights in the philosophy of science and on practice experience of interdisciplinary research collaboration aimed at the development of a medical technology, which can be used by experts in a particular discipline to analyse different elements of their discipline and, together with collaborators, to analyse the same elements from other disciplines. We believe that this systematic approach to understanding disciplinary perspectives will facilitate interdisciplinary research collaborations between experts from different fields. It will create awareness of one’s own disciplinary perspective and the ability to understand the disciplinary perspective of other experts at a sufficient level. Our framework thus aims to alleviate the challenge of *integration* in a collaborative research project by providing a tool for analysing *disciplinary perspectives*. We suggest that the concrete descriptions of disciplinary perspectives that result from the application of the framework, clarify the approaches of experts in a multi-disciplinary team. It thus enables effective communication through improved understanding of how each discipline contributes. Once researchers sufficiently understand each other’s discipline, they will be able to construct so-called conceptual models that integrate content relevant to the problems at hand.^4^

### Education in interdisciplinary research

In addition to professionals using our framework to facilitate collaboration in interdisciplinary research projects, we suggest that this framework can also be implemented in medical education. It can be used to teach students what it means to have a disciplinary perspective, and to explicate the role of disciplinary perspectives of disciplinary experts participating in an interdisciplinary research collaboration. We have implemented this framework in an innovative, challenge-based educational design that explicitly aims to support and promote the development of interdisciplinary research skills [22]. Research into the intended learning objectives has not yet been completed, but our initial findings indicate that the proposed framework effectively supports students in their ability to develop crucial skills for conducting interdisciplinary research projects. We suggest therefore that the framework can also be implemented in HPE as a scaffold for teaching and learning *metacognitive* skills needed in interdisciplinary research collaborations, for example between medical experts and engineers.

Research has shown that interprofessional education courses for healthcare students can have a positive effect on the knowledge, skills and attitudes required for interprofessional collaboration, but that organising such interventions is challenging [23, 24]. In the HPE literature, it is generally assumed that the limitations of interprofessional and interdisciplinary teamwork are due to problems of communication, collaboration and cooperation [25, 26], which are linked to barriers and enablers at institutional, organizational, infrastructural, professional and individual levels (e.g., [27, 28]). Therefore, interprofessional and interdisciplinary collaborations are discussed extensively in the HPE literature – our focus is challenges of interdisciplinary *research* collaboration.

The ability to use and produce knowledge and methods in solving (novel) problems is covered in the HPE literature by the notion of *adaptive expertise*, which encompasses clinical reasoning, integrating basic and clinical sciences, and the transfer of previously learned knowledge, concepts and methods to solve new problems in another context (e.g., [1, 29–34]). In previous work, we introduced the concept of interdisciplinary expertise, which expands on the notion of adaptive expertise by including the ability to understand, analyse and communicate disciplinary perspectives [8]. In this paper, we address the challenge posed by *how* this ability to understand, analyse and communicate disciplinary perspectives can be learned. The framework that we propose can be implemented in HPE to function as a tool to scaffold metacognitive skills of health professions students, facilitating the development of interdisciplinary expertise.

### Aims and contributions of this paper

Our first objective is to show that interdisciplinary collaboration in (medical) research faces not only institutional, but also cognitive and epistemological barriers. Therefore, we first provide a theoretical explanation of the concept of ‘disciplinary perspective’ as developed in the philosophy of science, in order to make it plausible that the cognitive barriers experienced by experts in interdisciplinary collaboration are the result of different disciplinary perspectives on a problem and its solution.

Our second objective is to provide a systematic approach to improve interdisciplinary research, for which we propose a framework, in the form of a series of questions, based on new insights from the philosophy of science into the epistemology of interdisciplinary research. We provide a detailed explanation of the application of the proposed framework in an interdisciplinary medical research project to illustrate its applicability in a multidisciplinary research collaborations, by showing that the different disciplinary perspectives that inform researchers and technicians within a multidisciplinary research team can be made transparent in a systematic way.

In short, our intended contribution is (i) to explain cognitive and epistemological barriers by introducing the concept of disciplinary perspectives in medical research collaborations, (ii) to offer a framework that enables the mitigation of these barriers within interdisciplinary research projects that are caused by different disciplinary perspectives, and (iii) to illustrate the applicability of this framework by a concrete case of an interdisciplinary research collaboration in a medical-technical research setting.

### Methods

We developed a framework for making disciplinary perspectives of experts participating in an interdisciplinary research collaboration explicit, by combining insights from the philosophy of science with practical experience from a medical research project. Philosophy of science provided the theoretical basis for our concept of disciplinary perspectives. Our detailed case-description stems from an interdisciplinary medical research project to develop and implement a new imaging tool for the diagnosis of kidney cancer, in which the first author participated. We then applied the framework to analyze and articulate the disciplinary perspectives of experts involved in this interdisciplinary medical research project.

The usefulness and applicability of the proposed framework was tested by the first author who, in her role as PI, was able to use it successfully in coordinating an interdisciplinary research project aimed at developing a biomedical technology for clinical practice [35, 36]. Below, we illustrate how the framework was systematically applied to this specific case, providing initial evidence of its applicability. However, to test whether the proposed framework reduces the cognitive and epistemological barriers caused by different disciplinary perspectives, experts need to be trained in its use. We suggest that training in the use of this framework requires, among other things, some insight into the philosophical underpinnings of the concept of ‘disciplinary perspective’. Our explanation of the so-called epistemology of disciplinary perspectives in this paper aims to provide such insight.

### Developing a framework for analysing and articulating a disciplinary perspective

The framework proposed here is based on insights about disciplinary perspectives in the philosophy of science. These insights concern an *epistemology* (a theory of knowledge) of scientific disciplines. In other words, the framework is based on an account of the knowledge-theoretical (epistemic) and pragmatic aspects that guide the production of knowledge and scientific understanding by a discipline [21].

The epistemology of scientific disciplines developed in our previous work is based on the philosophical work of Thomas Kuhn [37]. Building on his seminal ideas, we understand disciplinary perspectives as analysable in terms of a coherent set of epistemic and pragmatic aspects related to the way in which experts trained in the discipline (and who have thus, albeit implicitly, acquired the disciplinary perspective) apply and produce knowledge [38]. In our approach, the epistemic and pragmatic aspects that generally characterize a discipline, are made explicit through a set of questions that form the basis of the proposed framework (see Table 1, and the first column of Table 2). The disciplinary perspective can thus be revealed through this framework. In turn, when used in educational settings, this framework can be used to foster interdisciplinary expertise by acting as a scaffold for teaching and learning metacognitive skills for interdisciplinary research collaborations.^5^

**Table 1:** Framework for analyzing a disciplinary perspective

[1] What is the *overarching goal* of the (disciplinary) professional or research practice?
[2] What are the *kinds of phenomena* the discipline is typically interested in?
[3] What is the *objective of research* or investigation in the discipline (i.e., the objective of using and producing knowledge in this discipline)?
[4] What are the kinds of (mental or scientific) *models* or ‘*pictures’* to represent the knowledge about the phenomenon of interest?
[5] Which *theories* and *concepts* about the phenomena of interest are typically used in this discipline?
[6] Which *methodology* and (technological) *instruments* to explain or investigate the phenomena of interest are typically used in this discipline?
[7] What are the *practical constraints* regarding the overarching goal of the practice?
[8] Which are the *epistemic* and *pragmatic criteria* that the discipline aims to meet in using and producing (novel) knowledge about the phenomena of interest?

**Table 2:** Analysis by means of the framework (see Table 1) in the first column, of the disciplinary perspectives of four disciplines (I – IV) involved in the development of diffusion MRI for the diagnosis of kidney cancer. Next to the general descriptions of each disciplinary perspectives, each aspects is specified for the case at hand and relative to each discipline

Framework	I: Clinical practices	II: Medical biology	III: MRI physics & Diffusion MRI	IV: Signal and image processing
[1] What is the *overarching goal* of the (disciplinary) professional or research practice?	The *goal* of this practice is to diagnose and treat patients. In the case at hand, the goal is to diagnose and treat patients with kidney tumours according to their specific tumour type.	The *goal* of medical biology is to understand and describe (by visualizations, simple equations or words) mechanisms of human physiology and pathology. In the case at hand, the goal is to determine which tissue properties can be used to distinguish between kidney tumour tissue types (see I).	The *goal* of MRI physics is to develop machines that induce and manipulate the nuclear magnetic resonance (NMR) signal and to produce accurate representations of the object of imaging based on this signal. An example of the latter is visualizing microstructural properties of tissues by probing the diffusion signal. In the case at hand, the goal is to find those diffusion properties that correlate to tissue properties that can be used to distinguish between kidney tumour tissue types (see II).	The *goal* of signal and image processing is to turn NMR signals into visualizations and to process images so as to correct for artefacts and to derive relevant parameters (e.g., the diffusion coefficient). In the case at hand, the goal is to derive those parameters that reflect relevant diffusion properties to distinguish between kidney tumour tissue types (see III).
[2] What are the *kinds of phenomena* the discipline is typically interested in?	The *phenomenon of interest* is the disease, which includes how it can be diagnosed and treated. In the case at hand, it is the ‘kidney tumour’ as a clinical phenomenon, as something that affects a patient’s health. It includes how the tumour can be diagnosed, and whether it can be diagnosed by means of diffusion MRI (in addition to the standard clinical imaging and blood tests).	The *phenomena of interest* in the biomedical perspective on kidneys concern the functions and (dis)functioning of human physiology, divided in organs, systems of organs, organ components, cells, cell organelles, etc. In the case at hand, the organ of interest is the kidney, the tissue types that enables the kidney’s functioning and kidney tumour formation.	The *phenomena* that MRI physics deals with are magnetic fields, proton spins, radiofrequency pulses, relaxation times and coils.	The *phenomenon of interest* in signal and image processing is images as arrays of measurements, where each measurement is represented by a number. In other words, signal and image processing entail processing of arrays of numbers. This includes fitting mathematical equations, extrapolation, histogram analysis, noise suppression, etc.
[3] What is the *objective of research* or investigation in the discipline (i.e., the objective of using and producing knowledge) in this discipline?	The *objective of investigation* by a clinician is to diagnose the cause of a disease, and to develop a treatment plan. In the case at hand, the objective is to diagnose kidney tumours which involves determining its presence, type and severity of the disease and determining a suitable treatment plan.	The *objective of research* in medical biology is to describe and understand the *mechanisms* by which organs etc. fulfil their functions. The phenomena of interest (e.g., the functions or organs) are explained in terms of (micro) architecture, shapes (of organs or cells), molecules (e.g. proteins, hormones and Na+/K + pump) and interactions. In the case at hand, different tumour tissue types are described in terms of the presentation of their cells under the microscope (i.e., how they ‘look’) and in terms of their behaviour (i.e., how rapidly they grow).	The *objective of research* in this field involves developing new hardware (e.g., coils or other components of the machine) to improve image quality or to enable imaging of specific body areas, developing new acquisition sequences that allow new signal contrasts and hence visualizing new tissue aspects, and software development. In the case at hand, the objectives of investigation, and of technological advancement in diffusion MRI, firstly relate to developing models that accurately represent tissue physiology, and secondly, to apply diffusion-weighted imaging to different pathologies to investigate the feasibility of detecting changes in tissue structure due to these pathologies.	The *objective of research* in this field is to improve mathematical manipulations of the signals by developing better (e.g., more accurate to the imaged tissue) methods for fitting, extrapolation or analysis of the signal. In the case at hand, the objective is to develop algorithms that produce a combination of parameters that can be used to accurately characterize different kidney tumour types.
[4] What are the kinds of (mental or scientific) *models* or ‘*pictures’* build to represent the knowledge about the phenomenon of interest?	Clinicians construct a ‘picture’ of the specific patient and his/ her disease by gathering relevant but heterogeneous information, and fitting it into a coherent whole (the picture, or model of the patient, see Van Baalen & Boon 2015). The ‘picture’ of the patient enables further reasoning by the clinician about the patient, for instance, in order to get to a diagnoses, or to come up with a treatment plan that is suitable to the patient. In the case at hand, clinicians use their ‘picture’ of patients with different types of kidney tumours to explain how information that they can derive from what they see in a diffusion MRI scan will affect their clinical decisions regarding these patients.	The way of *modelling* in this disciplinary perspective is causal-mechanistic, that is, the phenomena of interest (functions) are described and explained by models that represent the causal-mechanisms. These models enable causal-mechanistic reasoning about the (dis)functioning of organs etc., in order to find causal explanations of the phenomena of interest. In the case at hand, these models enable understanding the mechanisms that can be used to explain the different tissue properties that can be used to distinguish between tumour types.	The behaviour of the phenomena of interest and their interactions are *modelled* through mathematical equations, some of which stem from basic physics (e.g., electricity and magnetism), while others are more specific to MRI, (e.g., related to relaxation times, and formulas describing the relationship between the acquired signal and the image, and equations relating the acquired diffusion NMR signal to diffusion properties of the tissue). These models enable reasoning, for instance, about how different tissue properties (e.g., tumour types) will result in different NMR signals.	The theoretical basis of this disciplinary perspective are mathematical models, such as the exponential model that is used to obtain the diffusion coefficient from the diffusion signal. These models are used to find the most appropriate (set of) diffusion parameters to characterize different kidney tumour tissue properties.
[5] Which *theories* and *concepts* (about the phenomena of interest) are typically used in this discipline?	In crafting a picture of a patient (or class of patients), clinicians use *medical concepts and theories*, including knowledge that concerns the diagnosis and treatment of a disease. The latter is mostly developed in clinical trials, studying the effectiveness and efficacy of treatments to specific patient populations, or the sensitivity and specificity of diagnostic tools. In the case at hand, clinicians specialized in kidney tumours are familiar with imaging of the kidney as a diagnostic tool to visualise the size and location of the kidney tumour.	*Theories and concepts* include theories that describe and explain the functions and mechanism of an organ or a system of organs. In the case at hand, an example is the mechanism with which blood is filtered in the glomeruli.	*Theories and concepts* that are fundamental to this field are classical electrodynamics (referring to (electro)magnetic fields, flux, induction, currents and coils), nuclear magnetic resonance (describing the magnetic moment of nuclei caused by resonance due to a magnetic field), relaxation and Fourier transform (fundamental to transforming the measured signal into images). Subfields are arranged according to acquisition method (e.g., diffusion MRI, functional MRI, etc.), body area or organ (e.g., abdomen, limbs, head, brain, heart) or pathology (e.g., tumours), or, focusing on technological advancement by developing new hardware or software development.	*Theories and concepts* in computer sciences (including mathematics) and information technology.
[6] Which *methodology* and (technological) *instruments* (to explain or investigate the phenomena of interest) are typically used in this discipline?	The *methodology* by which clinicians construct a picture of the patient (or a class of patients) is through gathering relevant information, on the one hand, by means of applying diagnostic techniques to the patient, and on the other hand, based on general medical knowledge and theory.	*Methodology* in medical biology include methods common in molecular biology, such as studying the cellular architecture of tissues and how molecules (proteins) are produced, excreted, metabolized and taken up by cells, in a laboratory environment (e.g., by looking at the organs themselves or the tissue in the microscope).	*Methods of investigation* in this field are, phantom experiments (to visualize or quantify known and controllable processes in MRI), experiments with healthy volunteers (to investigate the most adequate acquisition and modelling protocol for certain body areas), (clinical) trials with patient populations (to investigate the clinical value of a certain imaging protocol), and computer simulations (to simulate the effect of varying parameters).	The central *methodology* of image processing involves mathematical operations and computer programming, while experts in this discipline think of images as arrays and matrixes of numbers, rather than a literal representation of shapes and structures inside the body.
[7] What are the *practical constraints* regarding the overarching goal of the practice?	*Practical constraints* in constructing a picture of individual or classes of patients and in coming up with a treatment plan, are due to the workings of everyday clinical practice: doctors aim to diagnose and treat patients within the scarcity of time, knowledge and resources. In addition, treatment options are usually limited and associated with side effects. This implies that practical constraints are important to how clinicians assess diagnosis and treatment for specific (classes of) patients. In the case at hand, these practical constraints will play a role in how clinicians participating in the development of an MRI tool assess its diagnostic quality and usefulness.	*Practical constraints* in this field relate to the availability of instruments to observe or measure features of interest. The mechanisms medical biologists are interested in are not directly observable since they take place in a living body that cannot be taken apart to look at them. Hence, researchers have to come up with (indirect) measures and methods to manipulate cells, proteins and organs.	*Practical constraints* in this field concern *physical* aspects of the MRI apparatus (e.g., bore size, magnetic field strength, gradient strength), which limit the kinds of objects that can be imaged as well as the imaging parameters that can be obtained (e.g., field of view, signal to noise ratio, resolution). Another practical constraint is the *computing power* needed to transform the data into images.	An important aspect of image processing is the *available* software. Custom-made algorithms need to be programmed in general programming languages such as MATLAB and C++. Other *practical constraints* are primarily formed by the *computing power* of the computer with which data is transformed into images, but also by available software and scripts
[8] Which are the *epistemic and pragmatic criteria* that the discipline aims to meet in using and producing (novel) knowledge about the phenomena of interest?	*Epistemic criteria* playing a role in how a clinician uses and produces knowledge (i.e., how she produces the picture of a patient and how she uses it to reason about the patient and his disease) are *relevance* and *reliability* of information for the patient population and individual patients. In the case at hand, for example, the clinician takes into account knowledge about the incidence of different types of tumours related to age, gender, lifestyle, etc. Additionally, in assessing the new MRI tool, the clinician will take into account the relevance and reliability of images produced by it.	*Epistemic criteria* for knowledge production in medical biology is, firstly, that the mechanisms can *predict* and/or *explain* (i.e., have predictive and explanatory value) the phenomena of interest; secondly, that these predictions and/or explanations are *adequate*; and thirdly, that these mechanisms are *consistent* with other knowledge.	The *epistemic criteria* for knowledge production in this field are *consistency* between the theory, predictions and the phenomenon (the MRI signal) that is produced and measured; *reliability* of production and manipulation of the MRI signal, which is often established in terms of *reproducibility* (i.e., similar results are obtained for similar cases) and the *validity* of the images produced as representation for imaged object (i.e., a part of the human body).	Important *epistemic criteria* concern the *accuracy* of the models to the imaged tissue types, and the validity of the processed signal to the imaged object.

The general aspects indicated by italics in each question in Table 1 are interdependent, so that analysis using this framework results in a *coherent* description of the disciplinary perspective in terms of these aspects. The framework can be used by experts in an interdisciplinary research project not only to make explicit their disciplinary perspective in a general sense, but to also to *specify* in a systematic way how these aspects relate to the interdisciplinary research problem from their disciplinary discipline (see Table 2, which contains both the general and problem-specific descriptions for each aspect per discipline). In our view, this approach is productive in overcoming the cognitive and epistemological barriers. It thus contributes to productive interdisciplinary collaboration.

### Applying the framework in an interdisciplinary medical research project

To test the applicability of this framework, we applied it to an interdisciplinary medical research project. The interdisciplinary medical research project aimed at developing a new clinical imaging tool, namely, diffusion magnetic resonance imaging (i.e., diffusion MRI) to characterize the micro-structural makeup of kidney tumours, running from early 2014 to mid-2018. The first author was involved in this project as a principle investigator (PI). As an interdisciplinary expert with a background in *technical medicine*, which combines medical training with technological expertise [41], she coordinated and integrated contributions from experts with medical and engineering backgrounds. In her role as PI, she applied the proposed framework to analyse and articulate the disciplinary perspectives of other experts involved in the medical research project.

The aim of the interdisciplinary medical research project was to develop a new imaging tool for the characterization of renal tumours, i.e., diffusion MRI. Diffusion MRI allows for visualization and quantification of water diffusion without administration of exogenous contrast materials and is, therefore, a promising technique for imaging kidney tumours. In earlier studies, several parameters derived from diffusion MRI studies were found to differentiate between different tumour types in the kidney [42–44]. Existing imaging methods in clinical practice can detect the size and location of kidney tumours, but the tumour type and malignancy can only be determined histologically after surgery. The purpose of the medical research project was to assess whether more advanced parameters that can be obtained from diffusion MRI [35, 45] can differentiate between malignant and benign kidney tumours [36]. Being able to make this distinction could potentially prevent unnecessary surgery in patients with non-malignant tumours.

The interdisciplinary medical research project needed to bring together expertise (knowledge and skills) from different professionals, academic researchers as well as clinicians. Therefore, the research team consisted of a physicist, a biomedical engineer, a radiologist, a urologist and the principle investigator. The complex, interdisciplinary research object can be thought of as a system that encompasses several elements: the MRI-machine, the software necessary to produce images, the patient with a (suspected) kidney tumour, and the wider practice of care in which the clinical tool should function. In developing the clinical tool, these elements must be considered interrelated, whereas usually each expert focuses on one of these elements.

The PI utilized the framework to coordinate and integrate the contributions from different experts in the following manner. Throughout the project, she had meetings with each of the team members, where she probed them to explain their specific expertise in regard of the research object, as well as their expert contribution to the development of the imaging tool. Her approach in these meetings was guided by the general questions of the framework (Table 1). In this manner, she succeeded in getting a clear insight in aspects of each discipline relevant to the research object, and also in the specific contribution that needed to be made by each expert (as illustrated in Table 2 below). The level of understanding gained by this approach enabled her to, firstly, facilitate interdisciplinary team meetings in which disciplinary interpretations and questions from the experts about the target system could be aligned, and secondly, integrate their contributions towards the development of the new imaging tool [36].

In the presented approach, the framework was exclusively used by the PI, enabling her to acquire relevant information and understanding about the contributions of the disciplines involved. The other team members in the medical research project were not explicitly involved in applying the framework, nor in articulating their own disciplinary perspective or that of others. Hence, the resulting articulation of the disciplinary perspectives and of the contributions per discipline to the research object (in Table 2) is crafted by the PI. The level of understanding of the role of each discipline that the PI has acquired thereby appears to be sufficient to enable her coordinating task in this complex medical research project. Our suggestion for other research and educational practices, though, is that clinicians (as well as) other medical experts can develop this metacognitive skill by using the scaffold (in Table 1) in order to participate more effectively in these kinds of complex medical research projects.

In the results section we will first present our explanation and justification of the idea that disciplinary perspectives determine the specific approaches of experts (who have been trained in a specific discipline in using and producing knowledge) when faced with a complex problem. In this explanation and justification, we will use insights from the philosophy of science. Next, we will explain and illustrate the systematic use of the proposed framework (Table 1) by showing the results of applying it to the interdisciplinary medical research project.

## Results

The insights from philosophy of science on which the proposed framework for the explication of disciplinary perspectives is rooted in insights of the philosophers Immanuel Kant (1794–1804) and Thomas Kuhn (1922–1996). Their important epistemological insight was that ‘objective’ knowledge of reality does not arise from some kind of imprint in the mind, such as on a photographic plate, but is partly formed by the concepts and theories that scientists hold. These concepts and theories therefore shape the way they perceive the world and produce knowledge about reality. This philosophical insight provides an important explanation for the cognitive and epistemological barriers between disciplines. After all, scientific experts learn these concepts and theories by being trained within a certain discipline. In this way, they develop a disciplinary perspective that determines their view and understanding of reality. Based on this philosophical insight, we can imagine how these barriers can be bridged, namely by developing the metacognitive ability to think about their own cognition and how their scientific view of reality is shaped by their specific disciplinary perspective. In order to facilitate this ability, we develop a framework that can be used as a metacognitive scaffold. Finally, we apply this framework to an example interdisciplinary medical-technical research project, to illustrate it’s use in practice.

### Insights from the philosophy of science: disciplinary perspectives

Boon et al. (2019) refer to the notion of disciplinary perspectives and their indelible role in how experts approach problems —in particular, the ways in which experts use and produce knowledge in regard of the problem they aim to solve— and provide a philosophical account of this notion based on so-called constructivist (Kantian) *epistemology* (i.e., knowledge-theory, [38, 46]). On a Kantian view, ‘the world does not speak for itself,’ i.e., knowledge of (aspects of) the external world is not acquired passively on the basis of impressions in the mind (physically) caused by the external world (e.g., similar to how pictures of the world are physically imprinted on a photographic plate). Instead, the way in which people produce and use knowledge results from an interaction between the external world, the human senses and the human cognitive system. Crucially, neither our concepts nor our perceptions stem from passive impressions. Instead, ‘pre-given’ concepts ‘in the mind’ are needed in order to be able to perceive something at all and thus to produce knowledge about reality. Conversely, according to Kant, the imaginative (i.e. creative) capacity of the mind is then able to generate new concepts and to draw new connections of which the adequacy and usability must be tested against our experiences of reality. When new concepts (invented by the creative capacity of the human mind) have been tested against experience, they allow us to *see* new things in the external world, which we would not see without those concepts. This theoretical insight by Kant is crucial to get past naïve conceptions of knowledge, in particular, by understanding the indelible role of concepts in generating knowledge from observations and experiences.

This philosophical insight already makes it clear, for instance, that ‘descriptions of facts’ in a research project involve discipline-specific concepts, making these descriptions not easy to understand for someone who is not trained in that discipline. After Kant, this role of concepts has been expanded to the role of *perspectives*. For, Kuhn [37] created awareness that the human mind plays ‘unconsciously’ and ‘unintentionally’ a much greater role in the way scientific knowledge is created than usually assumed in the view that scientific knowledge is *objective*. Kuhn has introduced the concept of *scientific paradigm* to indicate in what sense the mind contributes. His idea was revolutionary because the notion of true and objective knowledge, which is the aim of science, became deeply problematic, as knowledge is only true and objective *within* the scientific paradigm, whereas it may even be meaningless in another.

Our notion of *disciplinary perspectives* is in many respects comparable to Kuhn’s idea of scientific paradigm, and is certainly indebted to Kuhn’s invention, particularly, with regard to the idea that it is a more or less coherent, usually implicit ‘background picture’ or ‘conceptual framework,’ which constitutes an inherent part of the cognitive system of an expert, and which forms the basis from which an expert thinks, sees and investigates in a scientific or professional practice. Furthermore, the scientific paradigm is not ‘innate,’ nor individually acquired, but maintained and transferred in scientific or professional practices, usually by being immersed in it. The same can be said about disciplinary perspectives. Yet, there are also important differences.

First, Kuhn believed that the paradigm is so deeply rooted in the cognitive structure of individual scientists, and, moreover, is embedded in how the scientific community functions, that it takes a *scientific revolution* and a new generation of scientists to shift into another paradigm, which is called a *paradigm-shift* (sometimes explained as a *Gestalt-switch*). Kuhn’s belief suggests that humans lack the capacity to reflect on their own paradigm.^6^ Conversely, we argue that humans can develop the *metacognitive ability* to perform this kind of reflection by which the structure and content of the paradigm or disciplinary perspective is made explicit. We take this as an important part of *interdisciplinary expertise*. Our suggestion, however, should not be confused with the idea that we can think without any paradigm or disciplinary perspective – we can’t, but we can explicate its workings (and adapt it), which is what we will illustrate in the case-description below.

Second, Kuhn’s focus was *science*, i.e., the production of *objectively true* scientific knowledge, in particular, theories. Instead, our focus is on *experts* trained in specific disciplines, who use and produce knowledge with regard to (practical) problems that have to be solved. Nonetheless, the Kuhnean insight explains why knowledge generated in distinct disciplines often cannot be combined in a straightforward manner (e.g., as in a jigsaw puzzle), which is due to the fact that knowledge is only fully meaningful and understandable relative to the disciplinary perspective in which it has been produced.

Our notion of *disciplinary perspectives* is similar to Kuhn’s idea of paradigm (which he specified later on as *disciplinary matrices*) in the sense that a paradigm functions as a *perspective* or a *conceptual framework*, i.e., a background picture within which a scientific or professional practice of a specific discipline is embedded and which guides and enables this practice. But instead of considering them as replacing each other in a serial historical order as Kuhn did, we assume that disciplinary perspectives co-exist, that is, exist in parallel instead of serial. This view on disciplinary perspectives can be elaborated somewhat further by harking back to Ludwik Fleck [47], a microbiologist, who already in the 1930s developed a historical philosophy and sociology of science that is very similar to Kuhn’s (also see [48]).^7^ Similar to and deeply affected by Kant, Fleck draws a close connection between human knowledge (e.g., facts) and cognition. Hence, Fleck disputes that *facts* are *descriptions* of things in reality discovered through properly passive observation of aspects in reality – which is why, according to Fleck, *facts* are *invented*, not *discovered*. Similar to Kuhn, Fleck expands on Kant by also including the role of the community in which scientists and experts are trained. Instead of *paradigms*, however, Fleck uses the terms *thought styles* and *thought collectives* to describe how experts in a certain professional or academic community adopt similar ways of perceiving and thinking that differ between disciplines: “The expert [trained in the discipline] is already a specially moulded individual who can no longer escape the bonds of tradition and of the collective; otherwise he would not be an expert” ([47], p. 54). But while Kuhn strove to explain radical changes in science, Fleck’s focus is on ‘normal science,’ that is, on communities (*thought collectives* each having their own *thought style*) that co-exist and gradually, rather than radically, change, which is closer to our take on disciplines. Importantly, according to Fleck, the community guides *which problems members of that communities find relevant* and how they approach these problems. Translated to our vocabulary, in scientific and professional practices, experts trained in different disciplines each have different disciplinary perspective, by means of which they recognize different aspects and problems of the same so-called *research object*, which they approach in accordance with their own discipline.

We propose that disciplinary perspectives can be analysed and made explicit, which we consider a crucial metacognitive skill of interdisciplinary experts. Our proposal for the framework to analyse disciplinary perspectives (in Table 1) takes its cue in Kuhn’s notion of disciplinary matrices. Kuhn’s original notion presents a matrix by which historians and philosophers can analyse the paradigm in hindsight, specifying aspects such as the metaphysical background beliefs and basic concepts, core theories, epistemic values, and methods, which all play a role in how knowledge is generated (also see [8, 50]). Our framework includes some of these aspects, but also adds others, thereby generating a scaffold that facilitates interdisciplinary collaborations aimed at applying and producing knowledge for complex problem-solving in professional research practices aimed at ‘real-world’ practices, such as medical research practice. Below, we will illustrate the application of this framework in a concrete case.

### Interdisciplinary research project: diffusion MRI for the diagnosis of kidney tumour

We will illustrate the applicability of the proposed framework (Table 1) for the analysis of disciplinary perspectives using the example of a research project that aims to develop a new clinical imaging tool, namely, diffusion MRI to characterize the microstructure of renal tumours. In our analysis, we focus on experts from four different disciplines: (I) clinical practice, (II) medical biology, (III) MRI physics, and (IV) signal and image processing. As indicated in the methods section, the complex, interdisciplinary research object that these experts have to deal with concerns a system consisting of the MRI-machine, the software necessary to produce images, and the patient with a (suspected) renal tumour, including the broader care practice in which the clinical tool should function.

In the following paragraphs we will first present a general explanation of the four disciplines involved in the project, and next, illustrate how the proposed framework can be applied to analyse and articulate each disciplinary perspective as well as the specific contribution of each discipline to the research object (in Table 2). It is not our intention to provide comprehensive descriptions of the fields that are involved, but rather to provide insight into how the fields differ from each other across the elements of our framework. In addition, we do not believe that all (disciplinary) experts only adhere to one disciplinary perspective. For example, clinicians usually combine both a clinical and biomedical perspective to fit together a complete picture of a patient for clinical decision-making concerning diagnosis and treatment [51–53]. Moreover, MRI engineers will usually need to combine insights from MRI physics and signal processing.

#### I. Clinical practice concerning patients with renal tumours

Clinical practice concerns the patient with a renal tumour. This practice differs from the other disciplines in our example, because it is not primarily a scientific discipline. Nonetheless, to develop a diagnostic tool, the disciplinary perspective of clinicians specialized in patients with kidney tumours is crucial, for example, to determine the conditions that the technology needs to meet in order to be useful for their clinical practice. The knowledge-base of clinical experts is rooted in biomedical sciences, which means that clinical experts often understand their patient’s signs and symptoms from a biomedical perspective (i.e., in terms of tumour formation of healthy renal physiology). Yet, clinicians will usually focus on their patient’s clinical presentation and possible diagnostic and clinical pathways. In clinical practice, several kidney tumour types are distinguished, each with its own histological presentation (visible under the microscope), tumour growth rate and chance of metastases. Unfortunately, all kidney tumour types, including non-malignant types, appear the same on standard imaging modalities, namely, as solid lesions. When the tumour is not metastasized, treatment consists of surgery removing the whole kidney or the part of the kidney that contains the tumour (i.e., ‘radical’ or ‘partial’ nephrectomy). If surgery is not possible, other treatments include chemotherapy or radiation. After surgery, a pathologist examines the tumour tissue to determine the tumour type. Occasionally, the pathologist concludes that the removed tumour was non-malignant, which is a situation that may be prevented if diffusion MRI can be used to distinguish between malignant and non-malignant tumours prior to surgery.

#### II. Medical biology

In biology, the structure and working of the body is studied at several levels, from the interaction of proteins and other macromolecules within cells to the functioning of organs. In the case at hand, the organ of interest is the kidney. Functions of the kidneys are excretion of waste materials, control of blood pressure via hormone excretion, balancing the body fluid, acid-base balance and balancing salts by excretion or resorption of ions. Understanding these functions requires insights into the anatomy, tissue architecture and physiology of the kidneys. The main functional structures of the kidney are: (1) the nephron, consisting of a tuft of capillaries (the glomerulus) surrounded by membranes that are shaped like a cup (Bowman’s capsule), responsible for the first filtration of water and small ions, and (2) the renal tubule that is responsible for more specific resorption and excretion of ions and water. The arrangement of small tubes that fan from the centre towards the outside (or cortex) of the kidneys allows maintaining variation in concentrations of ions, which helps to regulate resorption and excretion. The contribution of medical biology to the development of the diagnostic tool is important because knowledge about kidneys such as just sketched provides an understanding of the properties (i.e., microstructural of physiological properties) by which different tumour types can be distinguished from each other, which is crucial to interpreting the novel diagnostic imaging technology.

#### III. MRI physics & diffusion MRI

Magnetic resonance imaging is based on the physics of magnetism and the interaction of tissue components with radio magnetic fields. The main component of the human body that clinical MRI machines are sensitive to is (the amount of) water molecules or, more specifically, hydrogen nuclei (protons). These protons can be thought of as rotating or *spinning*, producing (tiny) magnetic fields. By placing tissue in a relatively strong magnetic field (usually 1.5 or 3 Tesla emitted by a large coil that surrounds the body), the tiny magnetic fields of protons (in the water-phase of the tissue) will align themselves with the direction of the strong magnetic field. By then applying a series of radiofrequency pulses, protons will be pushed out of balance and rotate back to their original state, causing a magnetic flux that causes a change in voltage which is picked up by receiver coils in the MRI machine. The rate with which protons return to their original state, the relaxation time, is influenced by the makeup of their environment, and will, therefore, differ for different tissues, resulting in image contrasts between tissues. To be able to form images of the signal, magnetic field gradients are applied, spatially varying the field which enables to differentiate between signals from different locations. Computer software using mathematical formulas ‘translate’ the signal into a series of images.

Diffusion MRI is a subfield of MR imaging, that is based on a contrast between ‘diffusion rates’ of water molecules in different tissues. Diffusion is based on the random (‘Brownian’) motion of water molecules in tissue. This motion is restricted by tissue components such as membranes and macromolecules and therefore water molecules move (or ‘diffuse’) at different rates in different tissues, depending on the microstructure of tissues. To measure this, additional magnetic field gradients are applied, which results in a signal attenuation proportional to the diffusion rate, as water molecules move (‘or diffuse’) out of their original voxel due to diffusion.

The method for acquiring diffusion-weighted images with an MRI machine (i.e., the ‘acquisition sequence’ of applying radiofrequency pulses and switching gradients on and off) is designed to gain sensitivity to the water molecules diffusing from their original location. The measured diffusion coefficient is considered to be related to microstructural properties of the tissue, namely the density of tissue structures such as macromolecules and membranes that restrict water diffusion. Together with other diffusion parameters that can be obtained by fitting the signal to other functions or ‘models’, the diffusion coefficient can be used to characterise and distinguish between different (tumour) tissue types, which is the aim of this new imaging tool.

#### IV. Signal and image processing

The signal acquired by MRI machines undergoes many processing steps before they appear as images on the screen. Some of these steps are performed automatically by the MRI system while others require standardized operations in the software package supplied by the manufacturer, and yet other, more advanced, manipulations are performed in custom-made programs or software packages developed for specific research purposes. In the field of diffusion MRI, software packages that perform the most common fitting procedures are available but often custom-made algorithms are required. The reason for this is that diffusion MRI is originally developed for brain imaging, while investigating its feasibility in other organs has started more recently and only makes up a small part of the field. New applications generate new challenges. For example, unlike the brain, kidneys (and other abdominal organs) move up and down as a consequence of breathing. Therefore, specific algorithms manipulating the scan to correct for this respiratory motion are required for diffusion MRI of the kidneys. Furthermore, as tissue structure and physiology in the kidneys differ from that in the brain, existing models need to be adjusted to that of the kidney.

## Discussion

In this paper, we have argued that interdisciplinary collaboration is difficult because of the role of experts’ disciplinary perspective, which shapes their view and approach to a problem and creates cognitive and epistemological barriers when collaborating with other disciplines. To overcome these barriers, disciplinary experts involved in interdisciplinary research projects need to be able to explicate their own disciplinary perspective. This ability is part of what is known as interdisciplinary expertise [8]. We defend that interdisciplinary expertise begins with creating awareness of the role of disciplinary perspectives in how experts view a problem, interpret it, formulate questions and develop solutions.

Analytical frameworks to guide interdisciplinary research processes previously developed by other authors typically focus on the *process* of interdisciplinary collaboration [9–15]. The approach we propose here contributes to this literature by addressing the deeper cognitive and epistemological challenges of interdisciplinary research collaboration on the role of the disciplinary perspective as an inherent part of one’s expertise [5, 16]. Several authors have already used the concept of ‘disciplinary perspectives’ to point out the challenges of interdisciplinary research (e.g., [9, 15]). Our contribution to this literature is the idea, based on philosophical insights into the epistemology of interdisciplinary research, that disciplinary perspectives can be made explicit, and next, to provide an analytical framework with which disciplinary perspectives within an interdisciplinary research context can be systematically described (as in Table 1) with the aim of facilitating interdisciplinary communication within such research projects.

Our further contribution is that we have applied this framework to a concrete case, thereby demonstrating that disciplinary perspectives within a concrete interdisciplinary research project can actually be analyzed and explicated in terms of a coherent set of elements that make up the proposed framework. The result of this analysis (in Table 2) shows a coherent description of the discipline in question per column, with an explanation per aspect of what this aspect means for the interdisciplinary research project. It can also be seen that the horizontal comparison (in Table 2) results in very different descriptions per aspect for each discipline. We believe that this example demonstrates that it is possible to explain the nature of a specific discipline in a way that is accessible to experts from other disciplines. We do not claim, therefore, that this table is an exhaustive description of the four disciplines involved. Instead, our aim is to show that the approach outlined in this table reduces cognitive and epistemological barriers in interdisciplinary research by enabling communication about the content and nature of the disciplines involved.

We suggest that educators can explore how the framework and philosophical underpinning can be implemented in HPE to support the development of students’ interdisciplinary expertise. Much has been written, especially in the engineering education literature, about the importance of interdisciplinarity and how to teach it. A recent systematic review article shows that the focus of education aimed at interdisciplinarity is on so-called soft skills such as communication and teamwork. Project-based learning is often used to teach the necessary skills, but without specific support to promote these skills [7]. In our literature review on education for interdisciplinarity [54–77], we did not find any authors who specifically address the cognitive and epistemological barriers to interdisciplinary collaboration as described in our article. One possible reason for this is that current epistemological views on the application of science in real-world problem-solving contexts, such as the research project presented here, do not recognise the inherent cognitive and epistemological barriers philosophically explained in this article [78]. The novelty of our approach is therefore our emphasis on the epistemological and cognitive barriers between disciplines that result from the ineradicable role of disciplinary perspectives in the discipline-bound way in which researchers frame and interpret the common problem. This makes interdisciplinary communication and integration particularly difficult. Specific scaffolds are needed to overcome these barriers. The framework proposed here, which systematically makes the disciplinary perspective explicit, aims to be such a scaffold. We therefore argue that much more attention should be paid to this specific challenge of interdisciplinary collaboration in academic HPE education. This requires both an in-depth philosophical explanation that offers a new view of scientific knowledge that makes clear why interdisciplinary research is difficult, and learning how to make disciplinary perspectives explicit, for which the proposed framework provides a metacognitive scaffold.

We have implemented this framework in a newly designed minor programme that uses challenge-based learning and aims to develop interdisciplinary research skills. In this minor, small groups of students from different disciplines work on the (interdisciplinary) analysis and solution of a complex real-world problem. A number of other scaffolds focused on the overarching learning objective have been included in the educational design, which means that the framework proposed here cannot be tested in isolation. Although our research into whether this new educational design achieves the intended learning goal is not yet complete, our initial experience of using the framework is positive. Students, guided by the teacher, are able to use the framework in their interdisciplinary communication - first in a general sense to get to know each other’s disciplines and then within their research project. This implies that the framework is useful in education aimed at learning to conduct interdisciplinary research.

This example, where the framework has been implemented in education aimed at developing interdisciplinary research skills, also shows that although it was developed in the context of a medical-technical research project, it is in fact very general and well suited for any interdisciplinary research.

A critical comment should be made regarding our preliminary evidence of the framework’s usefulness. The first author, who was PI of the interdisciplinary medical research project, in which she applied this framework in her role as coordinator, was also involved in the development of the framework [35, 36]. She, therefore has a detailed insight into the theoretical underpinnings of the framework in relation to its intended application. The lack of such a theoretical background may make it more difficult to apply the framework in interdisciplinary research.^8^ Which is why we have provided an extensive elaboration of these underpinnings in this paper.

Further research should address the question of whether this scaffold can facilitate interdisciplinary collaboration between disciplinary experts.

Further research is also needed to systematically analyse the value of this framework in HPE education. This starts with the question of what type of educational design it can be successfully implemented in. Other important questions are: Can interdisciplinary expertise be acquired without knowledge of the other discipline (e.g., biomedical engineering)? In other words, how much education in other disciplines should HPE provide to prepare experts to participate in specific interdisciplinary collaborations?

Furthermore, we emphasize that in addition to learning to use this framework as a metacognitive scaffold to gain a deeper understanding of the epistemological and cognitive barriers, students also need to develop other skills necessary for interdisciplinary research collaboration and working in interdisciplinary teams. The frameworks discussed in our introduction that analyse and guide the interdisciplinary research process provide insights into these skills (e.g. [9–12] and [54–77]).

We suggest that the article as a whole can be used in such educational settings to achieve several goals, provided that students are guided and coached by educators. First, to foster student’s understanding of the epistemological challenges of interdisciplinary collaboration and to recognize that these challenges are usually underestimated and not addressed in most approaches. Second, by providing insights into the epistemological challenges by outlining the philosophical underpinnings, students will be made aware of *having* a disciplinary perspective and how it guides their work. Finally, by providing a framework that can be used to analyse these disciplinary perspectives and by providing an example from the case description. When successful, this approach encourages students to developing transferrable skills that can be used in research projects beyond the initial educational project.

## Conclusions

Interdisciplinary research collaborations can be facilitated by a better understanding of how an expert’s disciplinary perspectives enables and guides their specific approach to a problem. Implicit disciplinary perspectives can and should be made explicit in a systematic manner, for which we propose a framework that can be used by disciplinary experts participating in interdisciplinary research projects. With this framework, and its philosophical underpinning, we contribute to a fundamental aspect of interdisciplinary collaborations.

## Data Availability

All data generated or analysed during this study are included in this published.

## Abbreviations

HPE: Health professions education
MRI: Magnetic Resonance Imaging
PI: Principle investigator

## Footnotes

1 In this article, we use ‘disciplines,’ ‘fields’ and ‘specialisms’ interchangeably.

2 Bridle (2013), Klein (1990), Newell (2007) and Szostak (2002) provide *activities* that are important for interdisciplinary collaborations, such as communication, negotiation and evaluating assumptions. In order to be able to perform such activities, students need to develop the appropriate skills [9, 17–19].

3 Roux et al. (2017) provide a clear characterization of transdisciplinary research: “A key aim of transdisciplinary research is for actors from science, policy and practice to co-evolve their understanding of a social–ecological issue, reconcile their diverse perspectives and co-produce appropriate knowledge to serve a common purpose.” ([20], p. 1).

4 Boon (2020, 2023) explains the notion of conceptual modelling in application oriented research [21, 22].

5 i.e., a framework that enables us to think analytically and systematically *about* our cognitive processes when we use and produce knowledge [39, 40].

6 Yet, we recognize that this belief was plausible in Kuhn’s era, where the idea that humans (including scientists) are inevitably and indelibly guided by paradigms and perspectives was revolutionary and devastating with regard to the rational view of man. But nowadays we have become familiar with this idea, which offers an opening for the metacognitive abilities that we suggest.

7 To scholars in HPE, we recommend the entry on Ludwik Fleck in the Stanford Encyclopedia of Philosophy [49].

8 The point made here touches on a more fundamental issue that is beyond the scope of this article. Namely, that resistance of students, but also of teachers, to the described approach may have to do with more traditional *epistemological beliefs* about science that do not fit well with the way scientific research works in practice [78, 79]. The philosophical underpinnings of the proposed framework explained in this article suggest alternative epistemological beliefs that are more appropriate for interdisciplinary research aimed at (complex) ‘real-world’ problems.
